# Anti-fatigue activity of methyl dihydrojasmonate and linalool in a rat model evaluated by a novel index for neuro-immune and oxidative stress interactions

**DOI:** 10.1038/s41598-024-60266-5

**Published:** 2024-05-09

**Authors:** Yasumitsu Nishimura, Kenta Nomiyama, Shuichiro Okamoto, Mika Igarashi, Yukino Sato, Hikaru Okamoto, Ayasa Kamezaki, Masumi Itadani, Futoshi Kuribayashi, Akira Yamauchi

**Affiliations:** 1 Department of Hygiene, Kawasaki Medical School, Kurashiki, 701-0192 Japan; 2 Shiono Koryo Kaisha, LTD, Osaka, 532-0033 Japan; 3 Department of Biochemistry, Kawasaki Medical School, Kurashiki, 701-0192 Japan

**Keywords:** Biochemistry, Immunology, Neuroscience, Physiology, Environmental sciences, Neurology

## Abstract

Avoiding fatigue is a long-standing challenge in both healthy and diseased individuals. Establishing objective standard markers of fatigue is essential to evaluate conditions in spatiotemporally different locations and individuals and identify agents to fight against fatigue. Herein, we introduced a novel method for evaluating fatigue using nervous system markers (including dopamine, adrenaline, and noradrenaline), various cytokine levels (such as interleukin [IL]-1β, tumor necrosis factor [TNF]-α, IL-10, IL-2, IL-5 and IL-17A), and oxidative stress markers (such as diacron-reactive oxygen metabolites [d-ROMs] and biological antioxidant potential [BAP]) in a rat fatigue model. Using this method, the anti-fatigue effects of methyl dihydrojasmonate (MDJ) and linalool, the fragrance/flavor compounds used in various products, were assessed. Our method evaluated the anti-fatigue effects of the aforementioned compounds based on the changes in levels of the nerves system markers, cytokines, and oxidative stress markers. MDJ exerted more potent anti-fatigue effects than linalool. In conclusion, the reported method could serve as a useful tool for fatigue studies and these compounds may act as effective therapeutic agents for abrogating fatigue symptoms.

## Introduction

Although fatigue is an unfavorable condition for the human body, it is unavoidable in modern life. Preventing or reducing fatigue has been a long-standing challenge for humans. Most fatigue symptoms are subjective; therefore, it is crucial to quantify fatigue with objective markers, representing the first step for fatigue treatment. Several studies have reported different objective markers for fatigue. For example, Aoki et al. reported the use of human herpesvirus (HHV)-6 and HHV-7 copy numbers in human saliva as markers of fatigue^1^. These standards can assist in comparing and evaluating the degree of fatigue even in spatiotemporally different locations and individuals. Fatigue is also a prominent symptom of several diseases such as chronic fatigue syndrome (also known as myalgic encephalomyelitis), neurological diseases, inflammatory diseases, and cancer^2,3^. Neurotransmitter catecholamines are reportedly altered in certain fatigue conditions; patients with chronic fatigue syndrome exhibited higher adrenaline and noradrenaline levels, heart rate, and tympanic temperature than control subjects^4^. Inflammatory cytokines in the aforementioned diseases are associated with fatigue symptoms. Interleukin (IL)-1β level increased in moderately ill patients with chronic fatigue syndrome^5^. Additionally, studies have shown that blocking IL-1 with anti-IL-1 antibodies alleviates fatigue in patients with rheumatoid arthritis^6^ and Sjögren’s disease^7^. Oxidative stress has also been associated with fatigue in both healthy people and patients with chronic fatigue syndrome^8^. In a human study, the levels of diacron-reactive oxygen metabolites (d-ROMs), a marker showing the accumulation of oxidized substances, increased in healthy people under acute stress (3 h-stress) and sub-acute stress (2 week-stress), and biological antioxidant potential (BAP), a marker reflecting anti-oxidative stress capacity, increased in healthy individuals under acute stress conditions^9^. Patients with chronic fatigue syndrome exhibited higher d-ROM and lower BAP levels than healthy people^9^.

Fragrances/flavors have been used for scent as well as for promoting physical and mental well-being^10^. Studies have been conducted to identify several functional compounds^11^. There are two main types of fragrances/flavors: natural extracts derived from plants and animals: and synthetic compounds developed artificially. Some fragrances/flavors, such as lavender, sweet orange, citrus, and peppermint extracts, are known to exert anti-fatigue effects^12^.

Linalool is a fragrance compound found in natural extracts such as lavender and coriander. Linalool is known to have several functional properties, including anti-inflammatory, anticancer, anti-hyperlipidemic, antimicrobial, antinociceptive, analgesic, anxiolytic, antidepressant, and neuroprotective properties^13^. Methyl dihydrojasmonate (MDJ), also known as Hedione^®^ or Claigeon™, is a derivative of jasmonate, a plant-based fragrant compound^14^. In recent years, MDJ has been reported to exert anti-tumor effects and is expected to be used as a functional fragrance^15–17^. Both MDJ and linalool can be artificially synthesized and are used as fragrance/flavor reagents in daily consumables. However, studies on the relationship between these functional fragrance/flavor compounds and fatigue, particularly their anti-fatigue effects based on quantitative biological markers, are lacking.

Therefore, in the present study, we aimed to evaluate the anti-fatigue effects of these compounds in a rat fatigue model, utilizing a novel marker to quantify the anti-fatigue effects and using nervous system markers, cytokine levels, and antioxidant markers. The anti-fatigue effects of both MDJ and linalool were assessed. These compounds may be useful therapeutic tools for alleviating fatigue symptoms.

## Results

### Establishment of the stress-load model and the effects of fragrance/flavor compounds

**Figure 1:**
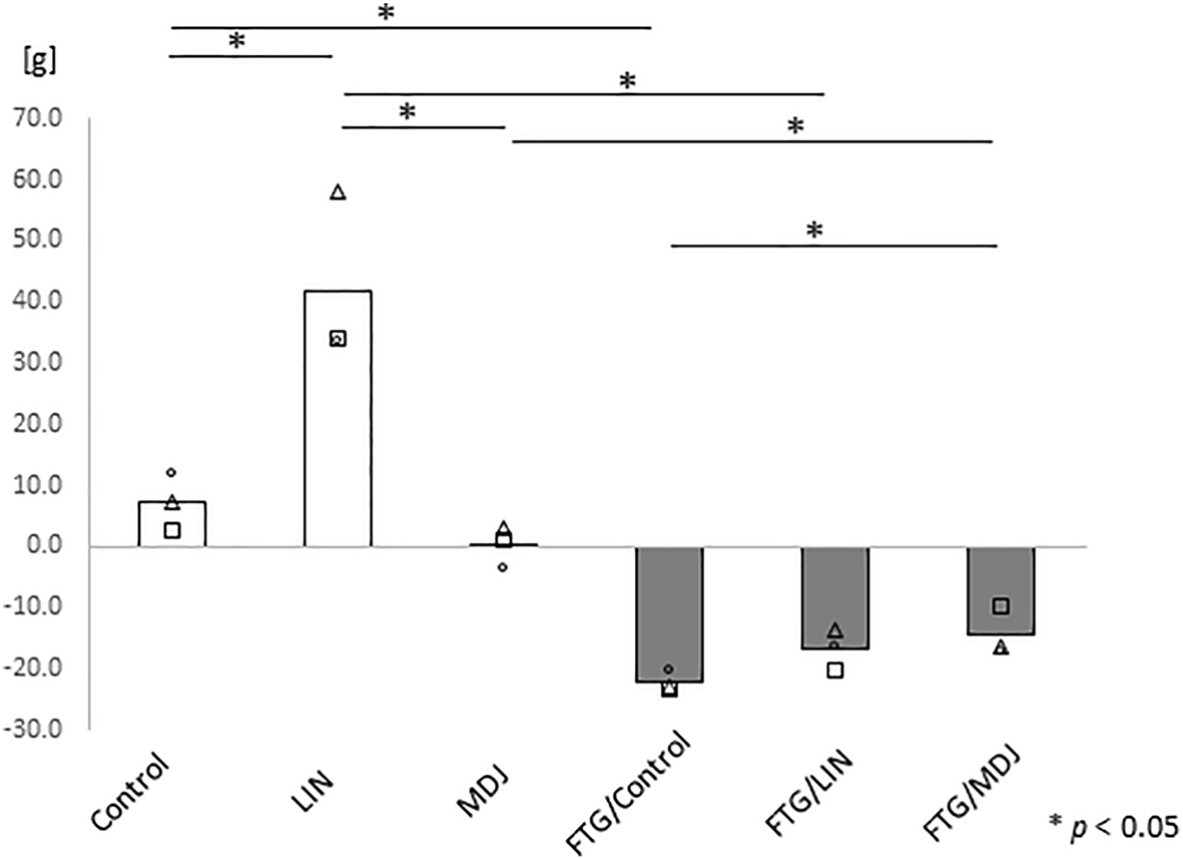
Changes in body weight with and without a stress challenge. Rats were housed in cages with or without a 1.5 cm depth of water. Each group was then divided into three subgroups: Control, LIN, or MDJ treatment. After a 24 h stress challenge, the body weight of each rat was measured. Each bar represents the mean value of the data and all the data points are plotted as circles, triangles and squares (n = 3). Mann–Whitney *U* test was performed. Control refers to no compound; LIN: linalool; MDJ: methyl dihydrojasmonate; FTG: fatigue by stress loading.

To evaluate the effects of linalool and MDJ on fatigue status, we adopted a water stress model in this experiment. Sprague–Dawley rats were placed in a cage filled with water up to a height of 1.5 cm for 24 h and deprived of sleep, which induced marked stress. During the stress-loading condition, the fragrance/flavor compounds linalool and MDJ were administered to rats (1 g/three rats/box). Thereafter, the body weight of the rats was measured to verify whether this model effectively induced stress and fatigue in the rats. All rats subjected to stress lost weight even though food and water were provided ad libitum. Gray bars in Figure 1 showed that this model was appropriate for inducing a fatigue condition. Rats under the fatigue condition with MDJ (shown as "FTG/MDJ") exhibited significantly less weight loss than those under the fatigue condition without MDJ exposure (shown as "FTG/Control"). Notably, most rats without fatigue or fragrance/flavor exposure (shown as "Control") exhibited slight weight gain, whereas those without fatigue condition with linalool (shown as "LIN") gained more weight than the control group.

### Effects of MDJ and linalool on the nervous and immune systems and oxidative stress

**Figure 2:**
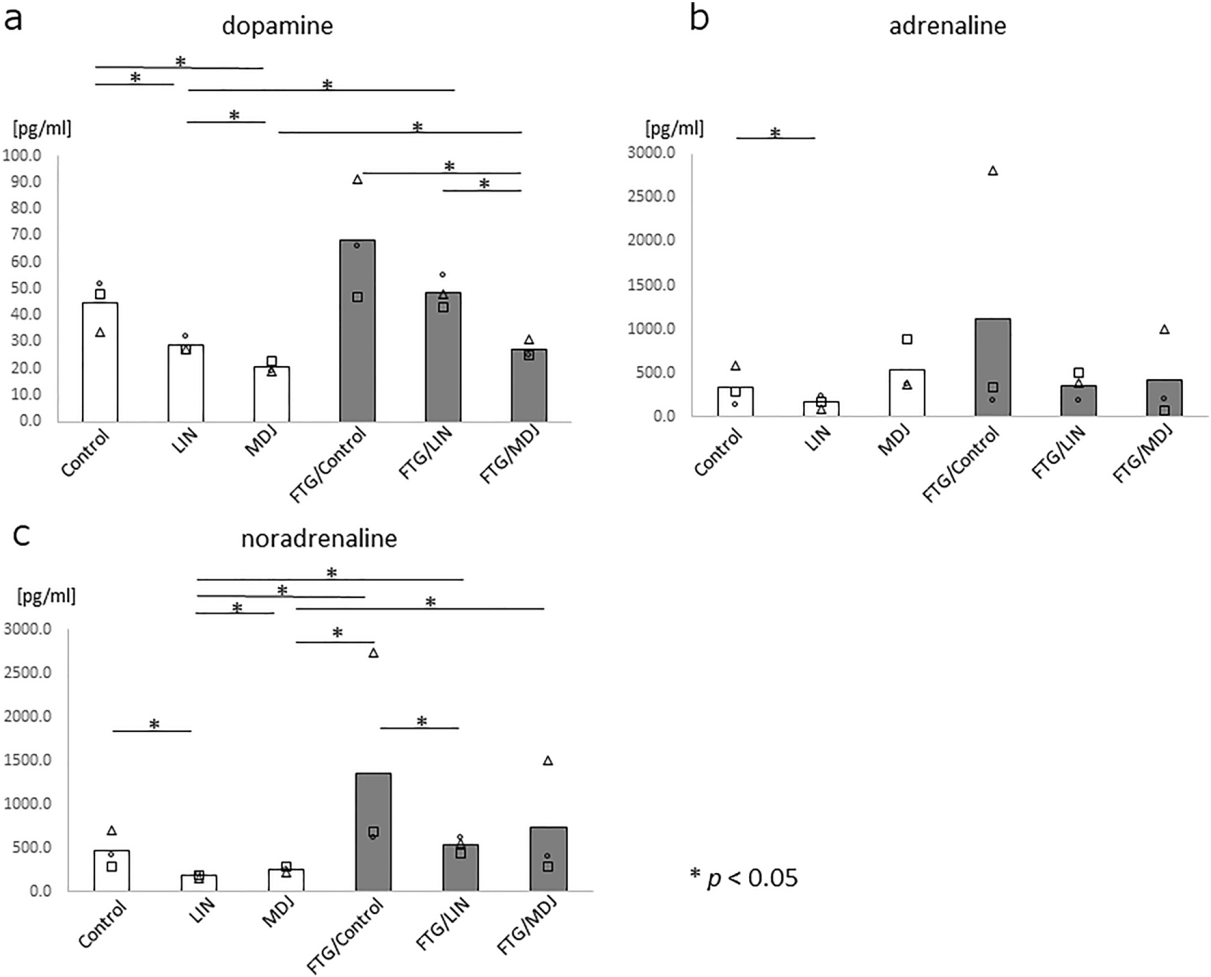
Catecholamines in peripheral blood with or without stress challenge. Rats were housed in cages with or without a 1.5 cm depth of water, and each group was further subdivided into three subgroups (Control, LIN, or MDJ treatment). After a 24 h stress challenge, peripheral blood was collected from each rat and catecholamine levels were measured. The levels of (**a**) dopamine, (**b**) adrenaline, and (**c**) noradrenaline in the peripheral blood of each rat are presented. Each bar represents the mean value of the data, and all data points are displayed as circles, triangles and squares (n = 3). The Mann–Whitney *U* test was performed. Control refers to no compound; LIN: linalool; MDJ: methyl dihydrojasmonate; FTG: fatigue by stress loading.

To determine the reason for the MDJ-induced reduction of weight loss during fatigue, various physiological parameters associated with the nervous and immune systems and oxidative stress were evaluated. To investigate the effects on the nervous system, we evaluated the levels of catecholamines, such as dopamine, adrenaline, and noradrenaline, in the peripheral blood and found that dopamine was significantly inhibited in rats exposed to MDJ under both fatigue and non-fatigue conditions (*p* < 0.05, Fig. 2a, MDJ and FTG/MDJ). Moreover, noradrenaline was inhibited in rats exposed to linalool under both the fatigue and non-fatigue conditions (*p* < 0.05, Fig. 2c, LIN and FTG/LIN). Adrenaline levels increased in the stress-loaded group without exposure (FTG/Control); however, the increase was not significant (Fig. 2b).

**Figure 3:**
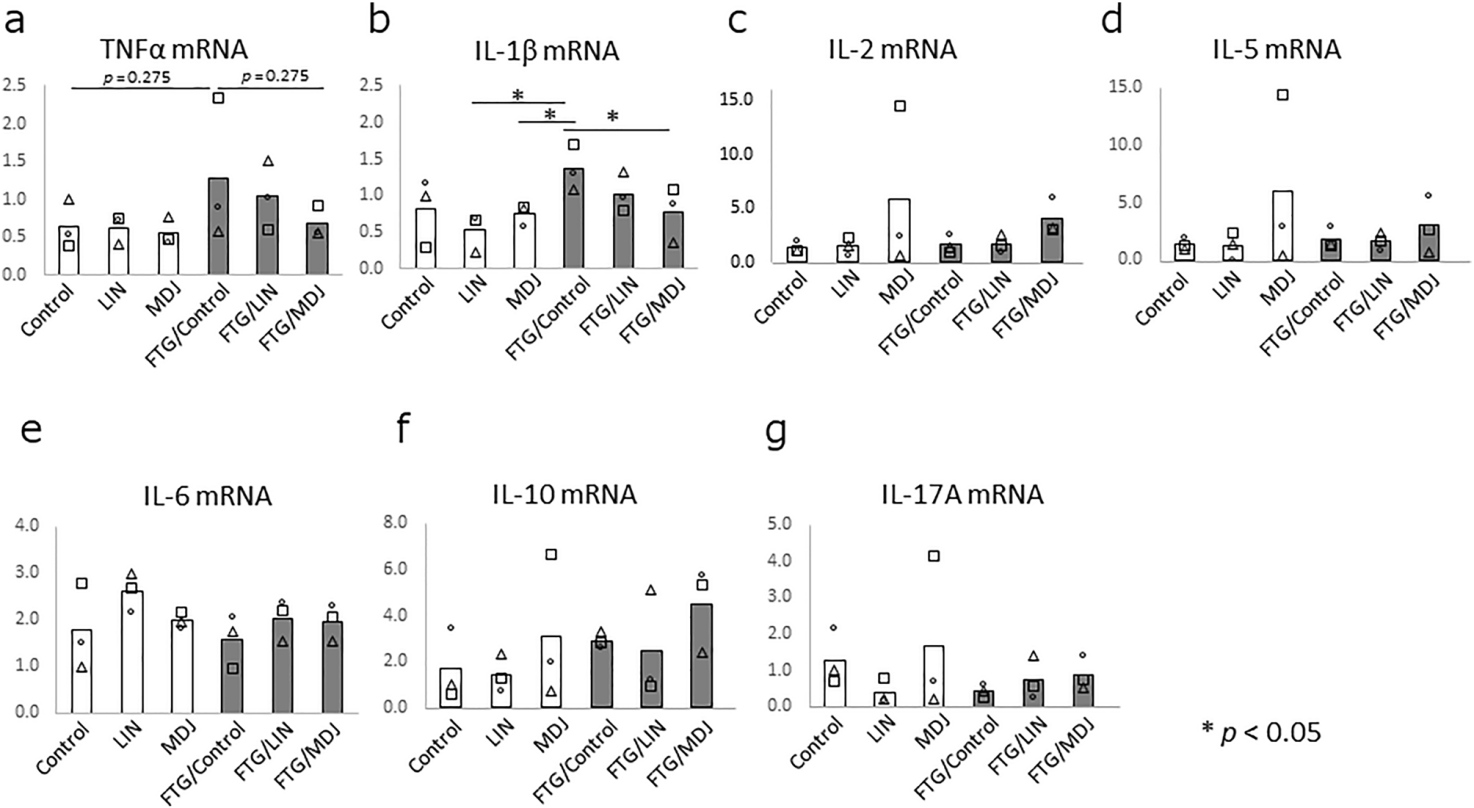
Messenger RNA (mRNA) expression levels of inflammatory cytokines in peripheral leukocytes. Rats were housed in cages with or without a 1.5 cm depth of water. Each group was then divided into three subgroups: Control, LIN, or MDJ treatment. After a 24 h stress challenge, peripheral blood was collected from each rat and leukocytes were isolated. Total RNA was extracted and complementary DNA was constructed from each sample. Quantitative PCR was performed to compare mRNA expression levels. (**a**) TNF-α, (**b**) IL-1β, (**c**) IL-2, (**d**) IL-5, (**e**) IL-5, (**f**) IL-10, and (**g**) IL-17A expression levels in peripheral leukocytes are displayed. Each bar represents the mean value of the data, and all data points are plotted as circles, triangles and squares (n = 3). Mann–Whitney *U* test was performed. Control: no compound; LIN: linalool; MDJ: methyl dihydrojasmonate; FTG: fatigue by stress loading.

Following this, the messenger RNA (mRNA) expression levels of inflammatory cytokines, such as tumor necrosis factor (TNF)-α, IL-1β, IL-2, IL-5, IL-6, IL-10, and IL-17A, in the peripheral leukocytes in the rats were assessed. Of these cytokines, TNF-α and IL-1β showed the tendency to increase in the stress-loaded rats (Control vs. FTG/Control, Fig. 3a,b, *p* = 0.275 and 0.127, respectively), and MDJ exposure in stress-loaded rats abrogated this increase in expression (FTG/Control vs FTG/MDJ, Fig. 3a,b, *p* = 0.275 and < 0.05, respectively). This indicates that MDJ exerts an inhibitory effect on stress-induced inflammation.

**Figure 4:**
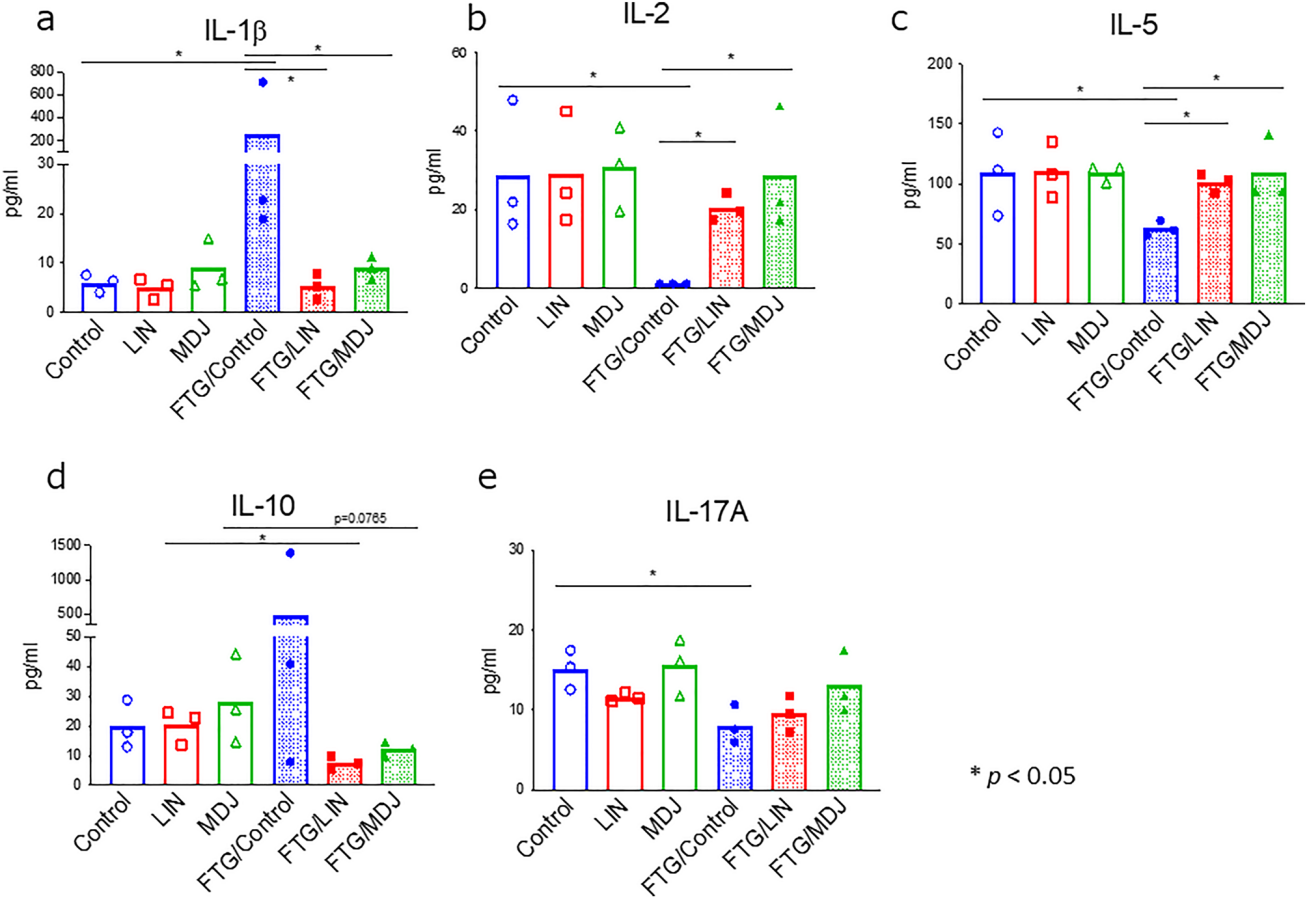
Levels of inflammatory cytokines in peripheral blood with or without stress challenge. Rats were housed in cages with or without a 1.5 cm depth of water. Each group was divided into three subgroups: Control, LIN, or MDJ treatment. After 24 h stress challenge, peripheral blood was collected from each rat and each rat Cytokine levels were determined using the Luminex method for (**a**) IL-1β, (**b**) IL-2, (**c**) IL-5, (**d**) IL-10, and (**e**) IL-17A expression levels. The mean value of each data set is represented by a bar, and all data points are shown (n = 3). The Mann–Whitney *U* test was performed. Control: no compound; LIN: linalool; MDJ: methyl dihydrojasmonate; FTG: fatigue by stress loading.

Subsequently, the concentration of cytokines in the serum was evaluated using peripheral blood to evaluate the immune system conditions using the Milliplex system (Merck Millipore, Burlington, MA, USA). We found that the concentrations of inflammatory cytokines IL-1β and IL-10 increased in rats in the fatigue condition; exposure to linalool and MDJ abrogated the increase in the concentrations of these cytokines (Fig. 4a,d). Additionally, IL-2, IL-5, and IL-17A, which are essential cytokines that promote the differentiation/proliferation of immune cells, were inhibited under fatigue conditions (FTG/Control, Fig. 4b, c, e), and exposure to linalool and MDJ abrogated the decrease in the concentration of IL-2 and IL-5 (FTG/LIN and FTG/MDJ, Fig. 4b,c).

**Figure 5:**
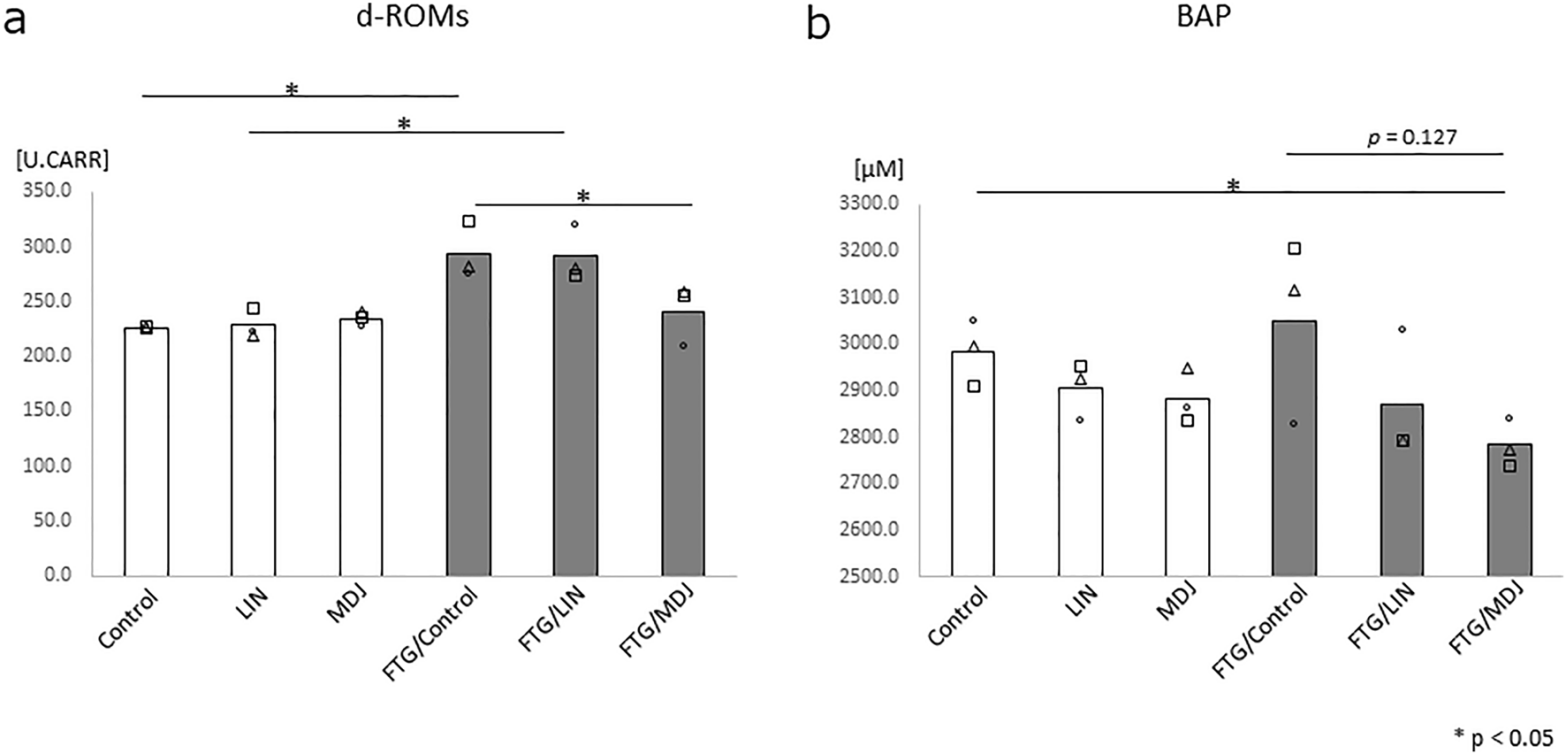
Oxidative stress markers in peripheral blood with and without stress challenge. Rats were housed in cages with or without a 1.5 cm depth of water. Each group was further divided into three subgroups: Control, LIN, or MDJ treatment. After 24 h stress challenge, peripheral blood was collected from each rat and d-ROMs and BAP levels were measured. (**a**) d-ROMs and (**b**) BAP values in peripheral blood are shown. Each bar represents the mean value and all data points are plotted (n = 3). The Mann–Whitney *U* test was performed. Control: no compound; LIN: linalool; MDJ: methyl dihydrojasmonate; FTG: fatigue by stress loading.

Additionally, we performed the d-ROM test, a marker showing the accumulation of oxidized substances, and the BAP test, a marker showing anti-oxidative stress capacity, to determine the status of oxidative stress in the stress-loaded rat model. In the d-ROM test, stress-loaded rats without fragrance/flavor exposure (FTG/Control) demonstrated significantly higher values than control rats without stress or fragrance/flavor exposure (*p* < 0.05; Fig. 5a). Stress-loaded rats with MDJ (FTG/MDJ) showed significantly lower d-ROM values than stress-loaded rats without any of the compounds (FTG/Control); the d-ROM values were similar to those in rats without stress (white bars; no significant difference was detected between the stress-loaded rats with MDJ and rats without stress). In contrast, the values from the BAP test between control rats with and without stress showed no significant changes. However, the values of the BAP test in stress-loaded rats with MDJ exposure tended to decrease (*p* = 0.127; Fig. 5b).

These data indicate that stress increases the levels of catecholamines, inflammatory cytokines, and oxidative stress and decreases the levels of immune-regulative cytokines, resulting in weight loss. Notably, MDJ alleviated most of these unbalanced parameters to a higher degree than linalool.

### Multivariate analysis of nervous and immune system parameters and oxidative stress

**Figure 6:**
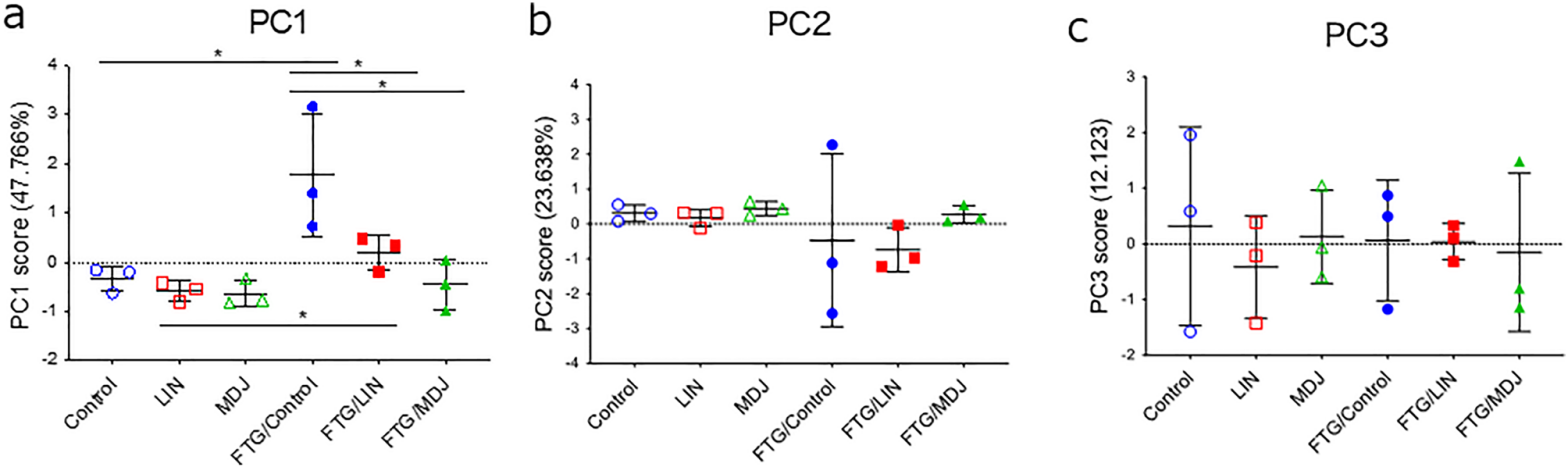
Multivariate analysis using parameters in neuro-immune-oxidative stress parameters. Nervous system parameters (dopamine, noradrenaline, and adrenaline in peripheral blood); immune system parameters (IL-1β and TNF-α mRNA, as well as IL-1β, IL-10, IL-2, IL-5, and IL-17A proteins in peripheral blood); and oxidative stress parameters (d-ROMs and BAP in peripheral blood) were analyzed in principal component analysis (PCA) in rats with or without stress challenge. (**a**) PC1, (**b**) PC2, and (**c**) PC3 represent the first, second, and third principal components, respectively. Asterisks (*) indicate statistical significance determined by Spearman’s correlation test. Control: no compound; LIN: linalool; MDJ: methyl dihydrojasmonate; FTG: fatigue by stress loading.

The results obtained from the experiments on the stress-load model indicated both significant and non-significant trends between the groups. Therefore, we investigated the relationships among all the examined parameters. It was found that plasma dopamine, adrenaline, and noradrenaline concentrations; d-ROM and BAP test values; IL-1β and TNF-α mRNA levels; and serum IL-1β and IL-10 concentrations showed positive correlations with each other, and serum IL-2, IL-5, and IL-17A concentrations also showed positive correlations with each other. In contrast, concentrations of IL-2, IL-5, and IL-17A were negatively correlated with dopamine and/or d-ROM levels (Table 1). These findings suggest that the aforementioned confirmed effects of MDJ on fatigue may be interconnected and comprehensively understood as an integrated score. Therefore, principal component analysis (PCA) was utilized to reduce the dimensions and extract the principal components (PCs) from the examined parameters. Three PCs, namely PC1 (contribution ratio 47.766%), PC2 (23.638%), and PC3 (12.123%) were extracted; in PC1, every parameter showed high or low factor loading of > 0.4 or < − 0.4 except for the TNF-α mRNA level (Table 2). PC1 showed positive values of factor loading for plasma dopamine, adrenaline, and noradrenaline concentrations; d-ROM and BAP test values; IL-1β mRNA level; and serum IL-1β and IL-10 concentrations, whereas it showed negative values for serum IL-2, IL-5, and IL-17A. Interestingly, the fatigue group without fragrance/flavor exposure (FTG/Control) showed a considerably higher calculated score of PC1 than the control group without fragrance/flavor exposure (Control), and the difference disappeared between the control and fatigue groups treated with linalool or MDJ (Fig. 6a). In contrast, PC2 and PC3 scores did not differ between the groups (Fig. 6b,c). Collectively, these findings suggest that MDJ alleviates fatigue by suppressing excess neuronal activity and inflammatory responses, and by increasing immune-activating cytokines to a higher degree than linalool.

**Table 1:** Statistical analyses for correlation across all examined parameters.

Spearman’s rho	DA	ADR	NA	d-ROM	BAP	IL-1β mRNA	TNF-α mRNA	IL-1β	IL-10	IL-2	IL-5	IL-17A
Dopamine (DA)	–	0.027	**0**.606**	**0**.513*	0.304	**0**.487*	0.271	0.171	− 0.199	− *0*.562*	− *0*.489*	− *0*.560*
Adrenaline (ADR)	0.027	–	**0**.501*	0.229	− 0.069	0.009	− 0.044	0.244	0.186	− 0.015	− 0.116	− 0.154
Noradrenaline (NA)	**0**.606**	**0**.501*	–	**0**.595**	0.098	**0**.595**	0.414	**0**.503*	− 0.129	− 0.33	− 0.324	− 0.452
d-ROM	**0**.513*	0.229	**0**.595**	–	0.065	**0**.533*	**0**.588*	0.357	− 0.209	− 0.388	− 0.414	− *0*.630**
BAP	0.304	− 0.069	0.098	0.065	–	0.191	0.082	0.092	**0**.619**	− 0.281	− 0.133	0.008
IL-1β mRNA	**0**.487*	0.009	**0**.595**	**0**.533*	0.191	–	**0**.692**	**0**.581*	0.064	− 0.248	− 0.184	− 0.362
TNF-α mRNA	0.271	− 0.044	0.414	**0**.588*	0.082	**0**.692**	–	0.32	− 0.142	0.086	0.069	− 0.387
IL-1β	0.171	0.244	**0**.503*	0.357	0.092	**0**.581*	0.32	–	0.42	− 0.265	− 0.296	− 0.298
IL-10	− 0.199	0.186	− 0.129	− 0.209	**0**.619**	0.064	− 0.142	0.42	–	0.076	0.096	0.243
IL-2	− *0*.562*	− 0.015	− 0.33	− 0.388	− 0.281	− 0.248	0.086	− 0.265	0.076	–	**0**.953**	**0**.623**
IL-5	− *0*.489*	− 0.116	− 0.324	− 0.414	− 0.133	− 0.184	0.069	− 0.296	0.096	**0**.953**	–	**0**.672**
IL-17A	− *0*.560*	− 0.154	− 0.452	− *0*.630**	0.008	− 0.362	− 0.387	− 0.298	0.243	**0**.623**	**0**.672**	–

Nervous system parameters (dopamine, adrenaline, and noradrenaline in peripheral blood); immune system parameters (IL-1β and TNF-α mRNA, as well as IL-1β, IL-10, IL-2, IL-5, and IL-17A proteins in peripheral blood); and oxidative stress parameters (d-ROMs and BAP in peripheral blood) were analyzed for correlation in rats with and without stress challenge. Each *p* value is described, and statistical significance is indicated by asterisks (* or ** for *p* < 0.05 or *p* < 0.01, respectively). Italics letters represent negative values, while bold letters represent positive values. DA; dopamine, ADR; adrenaline, NA; noradrenaline.

**Table 2:** Principal component analysis for all the parameters examined.

Parameter	Interpretation	Factor loading		
		PC1	PC2	PC3
		− 47.77%	− 23.64%	− 12.12%
Dopamine	Nervous system	**0**.**873**	0.012	− 0.014
Noradrenaline	Nervous system	**0**.**831**	**0**.**411**	0.077
Adrenaline	Nervous system	**0**.**739**	**0**.**592**	0.071
d-ROM	Oxidative stress	**0**.**648**	− **0**.**539**	0.190
BAP	Oxidative stress	**0**.**568**	− 0.124	0.348
IL-1β mRNA	Inflammation	**0**.**466**	− **0**.**542**	**0**.**543**
TNF-α mRNA	Inflammation	0.324	− **0**.**778**	**0**.**441**
IL-1β	Inflammation	**0**.**803**	**0**.**553**	0.125
IL-10	Inflammation	**0**.**791**	**0**.**565**	0.137
IL-2	Immune system	− **0**.**762**	0.293	**0**.**515**
IL-5	Immune system	− **0**.**697**	0.310	**0**.**555**
IL-17A	Immune system	− **0**.**575**	**0**.**553**	**0**.**435**

Nervous system parameters (dopamine, noradrenaline, and adrenaline in peripheral blood); oxidative stress parameters (d-ROMs and BAP in peripheral blood); inflammation parameters (IL-1β and TNF-α mRNA, as well as IL-1β and IL-10 proteins in peripheral blood); and immune system parameters (IL-2, IL-5, and IL-17A proteins in peripheral blood) obtained from rats with or without a stress challenge were analyzed using principal component analysis (PCA). PC1, PC2, and PC3 represent the first, second, and third principal components of the set, respectively. Bold colored cells indicate factor loading values over 0.4 or less than − 0.4

## Discussion

In this study, we analyzed the effects of fragrance/flavor compounds on various biological markers to prevent fatigue in a rat fatigue model and found that MDJ and linalool exhibited anti-fatigue effects based on neurological, inflammation/immunological, and oxidative stress markers. To the best of our knowledge, this is the first study to demonstrate that MDJ exerts a potent anti-fatigue effect. MDJ alleviated the abnormal status in stress-loaded rats in terms of their body weight; dopamine, IL-1β, IL-2, and IL-5 levels; and d-ROM value. Linalool also normalized noradrenaline, IL-1β, IL-2, and IL-5 levels in stress-loaded rats. Notably, MDJ was more effective than linalool in certain imbalanced parameters, such as suppressing weight loss (Fig. 1) and decreasing dopamine levels (Fig. 2A). Moreover, if these fragrances/flavors were ideal gases, based on the ideal gas equation of state (PV = nRT), the expected concentration would be 0.000039 mol/m^3^ and 0.0055 mol/m^3^ for MDJ and linalool, respectively (vapor pressure of MDJ and linalool: 0.095 Pa^18^ and 13 Pa^19^, respectively). It is believed that the actual concentration in this study was lower than the values mentioned. Moreover, this finding implies that MDJ demonstrated anti-fatigue effects at a notably lower concentration than linalool. In simpler terms, MDJ is considered to have a greater anti-fatigue effect than linalool.

We found that the combination of these neurological-immunological-oxidative stress markers such as dopamine, adrenaline, noradrenaline, d-ROMs, BAP, IL-1β, TNF-α, IL-10, IL-2, IL-5, and IL-17A correlated to fatigue condition through PCA. Using these neurological-immunological-oxidative stress markers may help in quantifying fatigue conditions more precisely and provide an objective assessment of anti-fatigue effects. These objective markers can be referred to as “stress-responding markers”. Of these markers, catecholamines, IL-1β, TNF-α, IL-10, and d-ROMs can be called “stress-activated markers”, which react to oppose stress. In contrast, IL-2, IL-5, and IL-17A can be called “stress-sensitive markers” which are suppressed by stress but are essential to immune reaction. Reducing “stress-activated markers” and increasing “stress-sensitive markers” may be a good strategy to fight fatigue. This approach would be beneficial for identifying functional substances with anti-fatigue effects such as fragrance/flavor compounds.

Both MDJ and linalool were found to exert an anti-fatigue effect in this study. However, they had different effects on body weight. MDJ suppressed weight gain in rats without stress and suppressed weight loss with stress, suggesting that it may be effective in maintaining a constant weight (Fig. 1). Linalool increased body weight in rats without stress (Fig. 1). A report suggests that the scent of lavender oil, which contains linalool as its main aroma, increases food intake^20^. Therefore, in this study, the appetite-stimulating effect of linalool may have resulted in temporary weight gain.

Neuro-immune interactions and oxidative stress have been studied for various disorders, such as chronic fatigue syndrome/myalgic encephalomyelitis, depression, rheumatoid arthritis, systemic lupus erythematosus, Sjögren’s disease, cancer, cardiovascular disorder, Parkinson’s disease, multiple sclerosis, and stroke^21^. Inflammatory cytokines are associated with disease activity and fatigue symptoms in these diseases. In rheumatoid arthritis and Sjögren’s disease, blocking IL-1 with antibodies alleviated fatigue symptoms^6,7^, serum TNF-α and IL-6 levels increased in patients with depression^22^, and serum IL-1RA, IL-6, TNF-α, and IP-10 levels increased in patients with cancer and fatigue^23,24^. In patients with multiple sclerosis, circulating TNF-α mRNA and TNF-α and interferon-γ protein levels increased^25,26^. In addition, treatment with anti-TNF-α antibody alleviated fatigue in sarcoidosis^27^. In the present study, MDJ and linalool normalized the values of these markers in a rat stress model, suggesting that MDJ, linalool, or fragrances/flavors containing these compounds may be effective in relieving fatigue symptoms in the aforementioned diseases. Further investigations, such as those on the underlying molecular mechanisms, are required to clarify this effect.

This study had some limitations that should be addressed. Herein, we examined three rats in each group, in order to minimize the number of animals used for testing for ethical reasons. Although we observed clear differences between the groups using PCA, the small sample size may have affected the statistical significance of singular parameters such as adrenaline, noradrenaline, and mRNA analyses (Figs. 2b,c, 3). Another limitation is that the results were confirmed only in the rat fatigue model. In order to apply these results in humans, it is necessary to demonstrate the anti-fatigue effects of MDJ and linalool in clinical studies.

In conclusion, we developed a novel assessment method to objectively quantify fatigue using a combination of neuro-immune interaction and oxidative stress parameters in a rat fatigue model. MDJ and linalool exhibited anti-fatigue effects in this model. The assessment method and fragrance/flavor compounds used in the present study could be helpful in preventing or alleviating fatigue.

## Methods

### Animals and stress exposure

All animal procedures were conducted in accordance with the ARRIVE guidelines (https://arriveguidelines.org) and the National Research Council’s Guide for the Care and Use of Laboratory Animals (https://grants.nih.gov/grants/olaw/guide-for-the-care-and-use-of-laboratory-animals.pdf) These procedures were approved by the Kawasaki Medical School Animal Experiments Committee’s ethics review (Number 18-116, 20-137, and 22-117) prior to the experimentation.

Male Sprague–Dawley rats (7-week-old) were purchased from CLEA Japan (Tokyo, Japan). Rats were housed in standard cages at 23 ± 1 °C, 50 ± 5% humidity, and 12 h bright–dark cycle and were fed autoclaved food and water ad libitum. To induce fatigue, rats were subjected to less sleep-induced stress by placing them in a cage with water up to a height of 1.5 cm for 24 h. Some rats were exposed to the fragrance/flavor compounds by housing the cage in a box (44 cm × 74 cm × 43 cm) containing volatilized linalool or MDJ at 1 g per three rats. Of the total 18 rats examined, half (nine) of them were kept under stress-free conditions, while the other nine rats were subjected to fatigue conditions. In each condition, three rats were not exposed to the fragrance/flavor compounds, and three rats were exposed to linalool or MDJ for 24 h. After exposure to stress and fragrance/flavor, the rats were anesthetized by inhaling 3–4% sevoflurane (Maruishi Pharmaceutical. Co., Ltd., Osaka, Japan) to prevent unnecessary excitement. Peripheral blood was collected through cardiac puncture using 2 mM ethylenediaminetetraacetic acid (Thermo Fisher Scientific Inc., Waltham, MA, USA). After centrifugation (750×*g*, 15 min at room temperature), the plasma and blood cell layers were collected. The plasma was frozen at − 20 °C and transferred for further examinations. Blood cells were processed for RNA extraction. Linalool and MDJ were purchased from commercially available sources.

### Evaluations of catecholamine levels

Plasma catecholamines were evaluated through high-performance liquid chromatography by SRL, Inc. (Okayama branch, Okayama, Japan), a clinical laboratory company, as an external contractor.

### Evaluations of cytokine levels

The levels of IL-1β, IL-10, IL-2, IL-5, and IL-17A in the serum were measured using a Milliplex system with Luminex200. The selected analytes were from the MILLIPLEX MAP Rat Cytokine/Chemokine Magnetic Bead Panel (Merck Millipore, Burlington, MA, USA) and the manufacturer’s protocols were followed for the assay. Each serum sample was incubated with mixed fluorescence beads with capture antibodies against IL-1β, IL-10, IL-2, IL-5, and IL-17A. Subsequently, the beads were incubated with biotinylated detection antibodies against these cytokines, followed by a reaction with phycoerythrin (PE)-labeled streptavidin. Finally, the median fluorescence intensity of PE was evaluated using Luminex200 for each analyte, and the concentration of each cytokine was calculated and defined using a standard curve of a five-parameter logistic fit.

### Quantification of RNA expression

Quantitative reverse transcription polymerase chain reaction (qRT-PCR) was performed using rat peripheral leukocytes. Briefly, total RNA was extracted from cells using an RNeasy kit (QIAGEN, Hilden, Germany). Template DNA was prepared with extracted total RNA of each sample using Ready-To-Go You-Prime First-Strand Beads kit (GE Healthcare, Little Chalfont, UK), and 0.5 μL each of the first strand DNA per sample was used for quantitative PCR (qPCR) with Fast SYBR Green Master Mix reagent (Life Technologies, Carlsbad, CA, USA). PCR signals were detected using Step One Plus Real-Time PCR System (Life Technologies). Primers used in this study are shown as presented in Table 3.

**Table 3:** Primers used to evaluate mRNA expression in this study.

Primer name		Sequence
rat* Il1b*	For	CAGGAAGGCAGTGTCACTCA
	Rev	TCCCACGAGTCACAGAGGA
rat *Il2*	For	CCCTGCAAAGGAAACACAGC
	Rev	CAAATCCAACACACGCTGCA
rat *Il5*	For	CTGTCCACTCACCGAGCT
	Rev	CTCTTCGCCACACTTCTC
rat *Il6*	For	AACTCCATCTGCCCTTCAGGAACA
	Rev	AAGGCAGTGGCTGTCAACAACATC
rat *Il10*	For	GCAGGACTTTAAGGGTTACTTGG
	Rev	GGGGAGAAATCGATGACAGC
rat *Il17a*	For	CTCAGACTACCTCAACCGTTCC
	Rev	CACTTCTCAGGCTCCCTCTTC
rat *Tnf*	For	AACTCGAGTGACAAGCCCGTAG
	Rev	GTACCACCAGTTGGTTGTCTTTGA
rat *Gapdh*	For	AACAGCAACTCCCATTCTTCC
	Rev	TGGTCCAGGGTTTCTTACTCC

For: forward; Rev: reverse.

### Evaluation of oxidative stress

To evaluate oxidative stress, d-ROM and BAP tests were performed. Frozen plasma samples were transferred, and d-ROM and BAP tests were performed by the Japan Institute for the Control of Aging, NIKKEN SEIL Co, Ltd. (Fukuroi, Shizuoka, Japan), a clinical laboratory company, as an external contractor.

### Statistical analysis

Statistically significant differences between two groups were examined using the Mann–Whitney *U* test. A *p* value less than 0.05 was considered to be statistically significant. The relationships among the parameters were assessed using Spearman’s correlation test to confirm the correlation coefficient values. To comprehensively examine the effect of MDJ on fatigue, PCA was conducted for all parameters. The calculated values of the extracted PCs were then statistically compared between the groups. All statistical analyses and PCA were performed using IBM SPSS Statistics version 28 (IBM, Armonk, NY, USA).

## Data Availability

All data generated or analyzed during this study are included in this published article.
